# Improving the microbial and physicochemical shelf life of yufka paste using *Lactobacillus plantarum* and calcium propionate

**DOI:** 10.1002/fsn3.3857

**Published:** 2023-11-27

**Authors:** Hannaneh Bahmanpour, Mahmood Sowti Khiabani, Sajad Pirsa

**Affiliations:** ^1^ Department of Food Science and Engineering, Faculty of Agriculture Islamic Azad University, Tabriz Branch Tabriz Iran; ^2^ Department of Food Science and Technology, Faculty of Agriculture University of Tabriz Tabriz Iran; ^3^ Department of Food Science and Technology, Faculty of Agriculture Urmia University Urmia Iran

**Keywords:** calcium propionate, food control, *Lactobacillus plantarum*, yufka bread

## Abstract

Considering the importance of reducing the use of chemical preservatives in food and the increasing attention of consumers to consume food products with minimal additives, the main goal of this research was to study the effect of use of chemical (calcium propionate) and biological (*Lactobacillus plantarum*) preservatives on increasing shelf life of yufka paste considering its physicochemical and microbial characteristics. For this purpose, three samples of yufka paste were prepared by adding concentrations of 10^3^, 10^5^, and 10^7^ cfu/mL of *L. plantarum* individually and three samples of paste were prepared by adding the same amount of bacteria in combination with 0.3% calcium propionate and these samples were compared with the control sample (without preservative) and the sample only containing 0.3% calcium propionate. The obtained results showed that different treatments and time had a significant effect on physicochemical properties including pH, moisture, and protein of yufka paste (*p* < .05). The results of the survival of *L. plantarum* showed that with increasing time, the survival rate of bacteria increased (*p* < .05). The pH of the samples showed that the *L. plantarum* has a significant effect on controlling the chemical quality of yufka during storage. The count of mold and yeast in the combined use of *L. plantarum* and 0.3% propionate was lower than the single use of propionate chemical preservative, which indicated the very good effect of the green preservative in controlling the moldy spoilage of yufka. Low concentrations of bacteria showed better antimold results than treatments containing bacteria and propionate in a combined form, propionate or control treatment.

## Graphical abstract

Yufka paste was prepared by adding concentrations of *Lactobacillus plantarum.* Microbial shelf life of yufka paste was improved using green preservative and calcium propionate. Green preservatives improved the sensory properties of the product while maintaining the physicochemical characteristics over time.

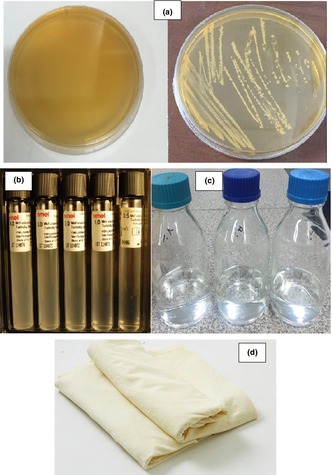

## 1 INTRODUCTION

Bread is one of the most important staple foods in the world and is generally considered a perishable commodity. Bread is very important in human nutrition as a source of protein and carbohydrates (Kourkouta et al., 2017; Shewry & Hey, 2015). Yufka, as semiprepared flatbread, is a paste with very little thickness, which is the birthplace of Turkey and is used in the preparation of the most delicious dishes of this country (Erkmen & Bozoglu, 2016; Sevgili et al., 2023). The ease of cooking and deliciousness of this product have made it one of the most popular foods in many European and Asian countries, and in some of these countries, this paste is known as phyllo. With this paste, you can prepare dozens of different types of food, desserts, sweets, and snacks such as samosas, tarts, pies, baklava, etc. Due to its low thickness, this paste dries quickly, so first of all, it must have the right storage conditions. It is better to keep this paste in the freezer, if possible, put it in nylon or zipped bags so that no air can reach it, otherwise the paste will become dry and brittle as a result (Levent & Bilgiçli, 2012; Pasqualone, 2018; Yıldız & Bilgiçli, 2015).

The most important problem of cereal products, especially types of bread, is the low shelf life due to contamination and moldy spoilage of these products during the storage period. The growth of mold in bakery products is very common and in many cases, the durability of the product is determined based on this. The decline in the quality of bread can be caused by several factors, the most common cause of which is microbial spoilage (Erkmen & Bozoglu, 2016). Fungal growth is the most common form of microbial spoilage in bread, which leads to huge economic losses as well as reduced safety for consumers due to the production of mycotoxins. Fungal spoilage of wheat bread is mainly caused by Penicillium species, which is the cause of spoilage of about 90% of bread made from wheat. Other bread‐spoiling fungi include Aspergillus, Monilia, Mucor, Endomyces, Cladosporium, Fusarium, and Rhizopus. The shelf life of bread without packaging is 3–4 days, and after this period, molds are noticeable on the surface of the bread, and with packaging, this period increases from 1 week to 1 month (Bartkiene et al., 2018; Garcia et al., 2019).

In recent years, the desire of consumers to use food without chemical preservatives has increased, and one of these methods of reducing chemical preservatives in food production is the use of biological preservatives. Biological preservative is the use of microorganisms or their metabolites. Lactic acid bacteria (LAB) play a key role in food fermentation, which not only contributes to the development of desired sensory properties in the final product but also increases their microbiological safety (Dopazo et al., 2023; Erkmen & Bozoglu, 2016; Le Lay et al., 2016; Sevgili et al., 2021). Natural preservatives such as essential oils, extracts, and food‐grade nanomaterials can be directly added to food products such as dairy products, types of bread, meat, etc., and can be used in the polymer structure of food product packaging. By delaying fungal spoilage, microbial spoilage, or oxidative spoilage, these substances can increase the shelf life of food crops and prevent waste of food products and costs (Daei et al., 2022; Pirsa, 2021; Shabkhiz et al., 2021; Yorghanlu et al., 2022).

Nowadays, the consumption of healthy and safe food is very important for human health. Considering the increasing desire of consumers to prepare healthy and natural foods with minimal processing and without preservatives, especially chemical preservatives, this research aims to investigate the effect of propionate and *L. plantarum* consumption on the microbial and physicochemical characteristics of yufka paste in order to improve the formulation and quality of this product.

## 2 MATERIALS AND METHODS

### 2.1 Chemicals

The wheat flour used to prepare yufka paste was Nol‐flour, which was obtained from Athar Flour Company (Tabriz, Iran). The specifications of the consumed flour include total fat 0.86 g/100 g, protein 8.202 g/100 g, carbohydrates 77.98 g/100 g, calcium 14.04 mg/100 g, and sodium 2 mg per 100 g. Sterilized water was used in all stages of product production. In order to prepare sourdough, instant bread yeast from Golmayeh Company (Tabriz, Iran) was used. This yeast was a combination of natural yeast (*Saccharomyces cerevisiae*) and emulsifier (sorbitan monostearate). Calcium propionate was obtained from Pasargad Novin Company (Tabriz, Iran). *L. plantarum* bacterium (ATCC 29521) was obtained from Tabriz University of Medical Sciences Pharmaceutical Research Center (Tabriz Pashmina Complex) as mother culture and to ensure its purity, it was cultured linearly on MRS agar culture medium. MRS agar, standard solutions of 0.5 McFarland, hydrochloric acid, silver nitrate, concentrated nitric acid, saturated potassium permanganate, saturated double ammonium ferric sulfate, ammonium thiocyanate, and other chemical compounds used were prepared and used from Merck (Germany).

### 2.2 Equipment and devices

The following devices and equipment with the mentioned specifications were used to perform chemical and microbial tests: digital scale (AND model), with an accuracy of 0.001 (Japan); Shimaz oven, with the ability to adjust the temperature from 50 to 250°C (Iran); Shimaz incubator, with the ability to adjust the temperature from 30 to 65°C (Iran); Sartreus Ohaus, model MB 25 (USA); spectrophotometer model DR 6000 (made in Germany); baking oven Ankara‐tech (Turkey); Kjeldahl device model 9840 k Hanon brand (China); electric oven (made in Korea); pH meter model pH 2211 made by Hana company (Italy); sampler in different sizes (10–1000 μL, made in Iran).

### 2.3 Preparation of raw materials to prepare yufka paste

#### 2.3.1 Preparation of bacterial suspension

**FIGURE 1:**
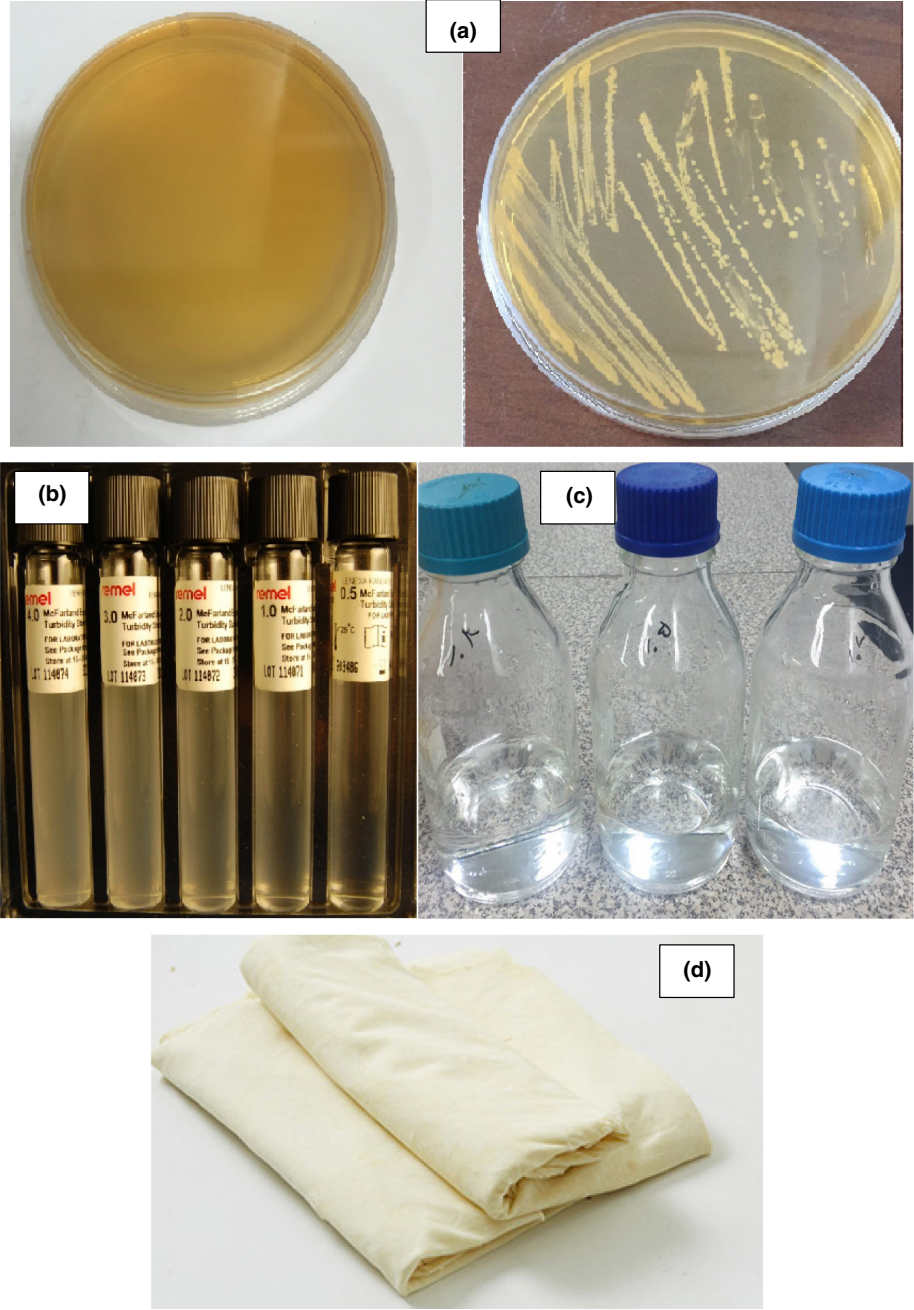
Linear culture on MRS agar culture medium for the purification of *Lactobacillus plantarum* (a), McFarland's standard solutions (b), and dilutions prepared from *L. plantarum* using the 0.5 McFarland method (c) and yufka paste sample prepared (d).

After preparing the *L. plantarum* (ATCC 29521), first, to ensure its purity, it was placed in a linear MRS agar culture medium under anaerobic conditions for 48 h at 30°C. Then, the grown colonies were stained. After this period, the culture medium containing bacteria was used to prepare bacterial suspensions in different dilutions (10^3^, 10^5^, and 10^7^ cfu/mL). For this purpose, 0.5 McFarland standard solutions were used. Next to the flame and under the hood (sterile conditions), 200 mL of physiological serum was poured into two bottles and some bacteria removed from the MRS agar culture medium with a loop and dissolved in the serum and after mixing was compared with a 0.5 standard in a spectrophotometer at a wavelength of 625 nm. In fact, 1 mL of 0.5 McFarland standard contains 10^8^ bacteria per milliliter, and its absorption in this wavelength is 0.08–0.13. Therefore, first, the dilution of 10^8^ cfu/mL of bacteria was prepared and then the dilutions of 10^3^, 10^5^, and 10^7^ cfu/mL were prepared serially and used in the next steps (Figure 1).

#### 2.3.2 Preparation of sourdough

Before preparing the desired treatments, sourdough was first prepared to reduce the possibility of bloating in yufka flat pastes due to the activity of *L. plantarum* bacteria. To prepare sourdough, water, flour, and yeast were mixed together and after initial mixing, the mixture was divided into three groups. In each batch, 125 mL of bacterial suspension with a certain number of colonies was added for 2.5 kg of dough. In fact, three dough samples were prepared and there were 10^3^, 10^5^, and 10^7^ bacterial colonies in each batch. These three batches of dough were kept in a greenhouse for 24 h at a temperature of 30–32°C.

### 2.4 Preparation of yufka paste samples

Pastes (treatments) were prepared using prepared sourdough. In order to prepare the control sample of yufka (control treatment), the paste containing flour (3 kg), salt (1.5%), and water (with a temperature of 30°C) was mixed using a mixer for 45 s. Then, the paste was made into a ball and by pouring flour on it to reduce stickiness, it was spread as thin as possible with a rolling pin and rollers, and then it was transferred to the oven and baked at a temperature of 280°C for 1 min (Levent & Bilgiçli, 2012). To prepare a sample of yufka paste with a chemical preservative (calcium propionate) (according to treatment number 7, Table 1), the paste including flour, salt, and water along with calcium propionate (70 g per 3 kg of paste) was mixed using a mixer for 45 s and baked at 280°C. In order to prepare yufka pastes with a biological preservative (*L. plantarum*) (treatment numbers 1–3 of Table 1), the paste was prepared by mixing raw materials including flour, salt, water, and sourdough containing bacteria (for 3 kg of paste 640 g of sourdough) using a stirrer for 45 s. Three batches of produced paste were spread with the help of rolling pin and roller until the thinnest possible and baking was done at 280°C for 1 min (Ryan et al., 2011). For the preparation of samples of yufka paste with a combined (chemical and biological) preservative (treatments of 4–6 in Table 1), the paste was prepared with a combination of raw materials including flour, salt, water, sourdough containing bacteria (640 g of sourdough for 3 kg of paste) and calcium propionate (70 g per 3 kg of paste) using a stirrer for 45 s. Three batches of produced paste were spread with the help of rolling pin and roller until the thinnest possible and baking was done at 280°C for 1 min (Figure 1) (Ryan et al., 2011).

**TABLE 1:** Treatments used to prepare yufka paste samples.

Treatment	Calcium propionate (%)	*Lactobacillus plantarum* (cfu/mL)	Time (Day)
1	0	10^3^	1, 5, 10, 15, 20
2	0	10^5^	1, 5, 10, 15, 20
3	0	10^7^	1, 5, 10, 15, 20
4	0.3	10^3^	1, 5, 10, 15, 20
5	0.3	10^5^	1, 5, 10, 15, 20
6	0.3	10^7^	1, 5, 10, 15, 20
7	0.3	0	1, 5, 10, 15, 20
Control	0	0	1, 5, 10, 15, 20

Eight prepared samples (one control sample, one sample containing calcium propionate, three samples containing *L. plantarum*, and three samples containing combined preservatives) were packed and coded in polyethylene bags after being cut into small pieces. The packages were kept at room temperature (25°C) for 20 days until further analysis. Each treatment was prepared in three repetitions and a total of 24 samples were examined. In the treatments containing sourdough, only mold counting was done because yeast growth will be observed due to the presence of yeast (*S. cerevisiae*).

### 2.5 Yufka paste tests

#### 2.5.1 Moisture

The moisture content of the samples was measured according to the national standard of Iran, No. 2705. For this purpose, 3 g of the sample was weighed with an accuracy of 0.001 g in plates that had already reached a constant weight, and the plates were placed in an oven at 103°C for 60 min. After the samples reached a constant weight inside the desiccator, the plates were weighed and the moisture content was calculated using the following equation.

(1) $$\text{Moisture}\;\left(\%\right)=\frac{M_{1}-M_{2}}{M_{1}}\times 100$$

where *M*_1_ is the weight of the sample before drying and *M*_2_ is the weight of the sample after drying.

#### 2.5.2 Salt measurement

In order to measure the salt of the prepared samples (Iranian national standard 6761), 2 g of the milled sample was accurately weighed and placed in a 250‐mL Erlenmeyer flask, and 10 mL of 0.1 normal silver nitrate solution was added to it, and then 10 mL of concentrated nitric acid was added and the mixture was heated to boiling. During boiling, 5 mL of saturated potassium permanganate was added to the solution, and boiling continued until the solution became colorless. Then, 100 mL of distilled water was added to it and it was titrated in the vicinity of the double ammonium sulfate reagent saturated with 0.1 normal ammonium thiocyanate solution until the appearance of a brick color, so that the created brick color remained stable for 15 s. The percentage of salt was calculated from the following equation:

(2) $$\text{Salt}\;\left(\%\right)=\frac{\left(V_{1}-V_{2}\right)\times 0.1\times 0.0585}{m}\times 100$$

where *V*_1_ is the volume of silver nitrate (mL), *V*_2_ is the volume of ammonium thiocyanate (mL), and m is the test weight (g).

#### 2.5.3 Measurement of insoluble ash in the acid

The basis of ash measurement is burning all organic matter and remaining mineral compounds and weighing it. This factor was measured using the 37 national standard method of Iran. For this purpose, 5 g of the samples was weighed with an accuracy of 0.001 g in crucible that had already reached a constant weight and were weighed, and the crucibles were baked in an oven with a temperature of 600°C for 4–6 h until the emergence of light gray color. After that, the crucibles were cooled in a desiccator at room temperature. Then, 25 mL of five normal hydrochloric acid was added to it and it was placed on a boiling bain‐marie with a closed lid for 10 min. Then, the solution was filtered with filter paper without ash and washed with water until complete washing of hydrochloric acid. The filter paper was returned to the same crucible, turned into ash in the furnace, and weighed in the desiccator after cooling. The percentage of ash insoluble in acid was calculated using the following equation.

(3) $$\text{Acid insoluble}\;\mathrm{ash}\;\left(\%\right)=\frac{\left(W_{1}-W_{0}\right)-\left({\overset{´}{W}}_{1}-{\overset{´}{W}}_{0}\right)}{W_{2}}\times 100$$

where *W*_1_ is the weight of the plant containing ash insoluble in sample acid (g), ${\overset{´}{W}}_{1}$ is the weight of the plant containing ash insoluble in filter paper acid (g), *W*_0_ is the weight of the empty test specimen (g), ${\overset{´}{W}}_{0}$ is the weight of the empty plant of filter paper test (g), and *W*_2_ is the sample weight (g).

#### 2.5.4 Measuring the pH

The pH was measured according to the national standard of Iran, No. 37. For this test, 10 g of the sample with 100 mL of boiled distilled water was mixed and let it sit for 20 min until it settles. Then, it was determined using a pH meter that was previously calibrated by standard buffer solutions.

#### 2.5.5 Measurement of the raw protein

The measurement of protein was carried out according to Iran's national standard 19052 using Kjeldahl apparatus. In this method, a specific amount of the sample was digested using sulfuric acid in the presence of a catalyst (Kjeldahl tablets include copper sulfate, titanium oxide, and potassium sulfate) for 2 h at a temperature above 400°C. The reaction product is digested, alkalized, and then distilled. Ammonia released in boric acid solution is collected and titrated with sulfuric acid solution in the presence of color indicator. In this way, the amount of nitrogen was measured and the amount of crude protein was calculated. The amount of crude protein in this method is calculated by multiplying the measured amount of nitrogen by the conversion factor according to the type of product, which is 5.7 in the case of ordinary wheat.

#### 2.5.6 *Lactobacillus plantarum* count

In order to evaluate the viability of *L. plantarum* during the storage period in different samples, samples were taken from the product. For this purpose, serial dilutions were prepared from the initial suspension. 0.1 mL of the desired dilutions were taken and cultured in MRS agar culture medium by pouring into a plate and kept in a greenhouse for 48 h at a temperature of 37–35°C. After this period, the bacteria were counted and reported as formed cells (cfu/g) (Erkmen, 2022).

#### 2.5.7 Counting the mold

According to National Standard of Iran No. 2‐10899 (2017), to determine these microorganisms in the produced samples, first prepare the selected culture medium and then by preparing certain dilutions (0.1 and 0.01) from the mixed and homogenized samples in the YGC culture medium cultured in the form of a pour plate and kept aerobically in a greenhouse for 5 days at a temperature of 25°C, and the results were expressed as the number of colony‐forming units per gram or milliliter of the product (Erkmen, 2022).

#### 2.5.8 Sensory evaluation

Sensory evaluation of the samples was done by 5‐point hedonic test by 20 untrained evaluators. The samples were provided to the evaluators at room temperature and coded. The investigated parameters included color and appearance, shape and symmetry, fragrance and flexibility, and overall acceptability. The grading method was as follows: very good = 5, good = 4, average = 3, bad = 2, and very bad = 1 (Basman & Köksel, 2001).

### 2.6 Modeling of microbial data

Growth analysis is considered as a valuable method in the quantitative investigation of growth and development and the number of molds. There are many regression models to describe sigmoid growth patterns. Logistic regression is a statistical regression model for dichotomous dependent variables such as disease or health, death, or life. This model can be considered as a generalized linear model that uses the logit function as the link function and its error follows a polynomial distribution. The Gomperts distribution is a continuous probability distribution that describes the life span distribution of adults with the help of demographics. Other scientifically related fields such as mold count and mold growth rate and biology also use Gompertz distribution for analysis instead of residuals (lives). In this study, nonlinear regression models commonly used in growth analysis studies (logistic and Gompertz) were used to describe the number of molds (Rodríguez‐Saavedra et al., 2021; Schvartzman et al., 2014).

In order to check the microbial results and predictability of the mold count observed on yufka pastes, the modeling method was used and the models of Table 2 were presented. Modeling of the obtained data was done using the investigated models according to the equations by MATLAB software (Table 2).

**TABLE 2:** Models used in MATLAB software.

Model	Equation	Modified equation
Logistic	$y=\frac{a}{\left[1+\exp\left(b-\mathrm{cx}\right)\right]}$	$y=\frac{A}{\left\{1+\exp\left[\frac{4\mu_{m}}{A}\left(\lambda -1\right)+2\right]\right\}}$
Gompertz	$y=a.\exp\left[‐\exp\left(b-\mathrm{cx}\right)\right]$	$y=A\exp\left\{‐\exp\left[\frac{\mu_{m.e}}{A}\left(\lambda -\iota \right)+1\right]\right\}$

### 2.7 Statistical analysis

The factorial in the form of a completely randomized design with the control sample and seven treatments (including propionate and different concentrations of *L. plantarum*) (Table 1) was used to prepare the different yufka samples. All the tests were performed for 20 days with 5‐day intervals (1st day, 5th day, 10th day, 15th day, and 20th day) and in three repetitions. Statistical analysis of data was done with SPSS 16 software and comparison of means was done using Duncan's multirange test at 95% probability level. Also, Matlab version 2009 software was used for data modeling. Graphs were drawn in Excel 2010.

## 3 RESULTS AND DISCUSSION

### 3.1 Moisture, salt, and ash

Table 3 shows the comparison of the average results of moisture content, salt percentage, and ash amount in different treatments of yufka pastes during storage time. The results of analysis of variance of the data related to the moisture content of yufka pastes showed a significant effect of the treatments in different concentrations (*p* < .05). Also, the time analysis showed that the humidity changed significantly during the storage time (*p* < .05). Other studies have been conducted in the field of using sourdough on the moisture content of prepared breads. Bolourian et al. (2010) have shown that as the amount of bacteria increases and time increases, the amount of moisture decreases. Abu‐Ghoush et al. (2008) also stated that different concentrations of chemical preservatives did not have a significant effect on the moisture level of flat paste, but the passage of time up to 3 days decreased the moisture level of bread. These findings are in accordance with the results obtained in Table 3, which indicate a significant decrease in moisture content during storage (Bolourian et al., 2010). That is, with the increase of shelf life, the moisture content of yufka pastes decreases. It can also be due to the loss of moisture during the decrease in humidity. The results of the analysis of variance of the data related to the amount of salt in the samples showed a significant effect of the treatments in different concentrations (*p* < .05) and a significant effect of the storage time on the salt content of the pastes (*p* < .05). According to the comparison of average data by Duncan's method, the amount of salt in the paste samples increases over time. Due to the fact that the moisture content of paste decreases during the storage period; therefore, the measured salt concentration increases, also in the treatments in which propionate salt is used, the salt concentration is higher than in other treatments. The results of the analysis of variance of the data on the amount of ash insoluble in acid showed that treatments in different concentrations and storage time did not have a significant effect on the amount of ash insoluble in acid (*p* > .05). Table 3 also shows the comparison of average data by Duncan's method during the storage period, and as can be seen in the table, different treatments on different days did not show statistically significant differences.

**TABLE 3:** Comparison of the average results of moisture content, salt percentage, and ash amount in different treatments of yufka paste during storage time.

Treatment	Characteristics	Day 1	Day 5	Day 10	Day 15	Day 20
1	Moisture	22.57 ± 0.36^BCab^	21.98 ± 0.28^Cab^	22.14 ± 0.48^BCab^	22.01 ± 0.66^ABab^	21.51 ± 0.73^Aab^
	Salt	0.41 ± 0.09^Abc^	0.52 ± 0.06^BCbc^	0.54 ± 0.11^BCbc^	0.63 ± 0.07^Cbc^	0.44 ± 0.13^ABbc^
	Ash	0.05 ± 0.01^Aa^	0.05 ± 0.01^Aa^	0.06 ± 0.02^Aa^	0.05 ± 0.00^Aa^	0.06 ± 0.01^Aa^
2	Moisture	23.26 ± 0.62^BCc^	22.15 ± 0.43^Cc^	19.94 ± 0.18^BCc^	20.51 ± 1.38^ABc^	20.35 ± 0.19^Ac^
	Salt	0.49 ± 0.10^Abc^	0.68 ± 0.06^BCbc^	0.53 ± 0.03^BCbc^	0.56 ± 0.15^Cbc^	0.42 ± 0.07^ABbc^
	Ash	0.04 ± 0.01^Aa^	0.05 ± 0.01^Aa^	0.04 ± 0.00^Aa^	0.05 ± 0.00^Aa^	0.04 ± 0.01^Aa^
3	Moisture	24.66 ± 0.45^BCa^	22.99 ± 0.59^Ca^	22.32 ± 1.01^BCa^	23.02 ± 1.19^ABa^	21.99 ± 0.88^Aa^
	Salt	0.55 ± 0.144^Aabc^	0.48 ± 0.21^BCabc^	0.50 ± 0.05^BCabc^	0.56 ± 0.04^Cabc^	0.66 ± 0.09^ABabc^
	Ash	0.05 ± 0.01^Aa^	0.06 ± 0.01^Aa^	0.04 ± 0.00^Aa^	0.06 ± 0.00^Aa^	0.05 ± 0.00^Aa^
4	Moisture	22.50 ± 0.66^BCab^	23.25 ± 0.86^Cab^	22.37 ± 0.45^BCab^	21.59 ± 1.63^ABab^	21.68 ± 0.10^Aab^
	Salt	0.61 ± 0.05^Abc^	0.54 ± 0.04^BCbc^	0.60 ± 0.02^BCbc^	0.57 ± 0.07^Cbc^	0.59 ± 0.26^ABbc^
	Ash	0.06 ± 0.01^Aa^	0.06 ± 0.01^Aa^	0.04 ± 0.00^Aa^	0.05 ± 0.00^Aa^	0.04 ± 0.01^Aa^
5	Moisture	24.06 ± 0.62^BCc^	23.23 ± 0.48^Cc^	20.99 ± 0.17^BCc^	21.95 ± 0.82^ABc^	19.79 ± 1.19^Ac^
	Salt	0.59 ± 0.15^Ac^	0.42 ± 0.04^BCc^	0.54 ± 0.05^BCc^	0.66 ± 0.06^Cc^	0.73 ± 0.04^ABc^
	Ash	0.05 ± 0.01^Aa^	0.04 ± 0.00^Aa^	0.06 ± 0.02^Aa^	0.05 ± 0.02^Aa^	0.04 ± 0.00^Aa^
6	Moisture	24.10 ± 0.43^BCc^	24.02 ± 0.23^Cc^	22.61 ± 0.44^BCc^	19.03 ± 1.60^ABc^	19.03 ± 0.51^Ac^
	Salt	0.56 ± 0.08^Abc^	0.68 ± 0.05^BCbc^	0.62 ± 0.11^BCbc^	0.53 ± 0.09^Cbc^	0.65 ± 0.07^ABbc^
	Ash	0.04 ± 0.00^Aa^	0.06 ± 0.01^Aa^	0.05 ± 0.00^Aa^	0.04 ± 0.00^Aa^	0.04 ± 0.00^Aa^
7	Moisture	22.96 ± 0.25^BCc^	21.36 ± 0.60^Cc^	21.13 ± 1.67^BCc^	20.56 ± 0.65^ABc^	20.02 ± 0.59^Ac^
	Salt	0.64 ± 0.05^Aab^	0.49 ± 0.06^BCab^	0.52 ± 0.05^BCab^	0.75 ± 0.25^Cab^	0.77 ± 0.05^ABab^
	Ash	0.04 ± 0.01^Aa^	0.04 ± 0.00^Aa^	0.05 ± 0.01^Aa^	0.05 ± 0.00^Aa^	0.06 ± 0.01^Aa^
Control	Moisture	23.08 ± 0.46^BCb^	22.89 ± 0.33^Cb^	23.19 ± 0.45^BCb^	21.59 ± 1.26^ABb^	22.01 ± 0.26^Ab^
	Salt	0.40 ± 0.03^Aa^	0.50 ± 0.04^BCa^	0.48 ± 0.05^BCa^	0.53 ± 0.21^Ca^	0.54 ± 0.20^ABa^
	Ash	0.05 ± 0.01^Aa^	0.06 ± 0.01^Aa^	0.05 ± 0.00^Aa^	0.06 ± 0.01^Aa^	0.05 ± 0.00^Aa^

*Note*: Similar lowercase letters depending on the treatment (row) and uppercase letters depending on the time (column) indicate no significant difference at the 5% probability level.

### 3.2 pH and protein

Table 3 shows the comparison of the average results of pH and protein in different treatments of yufka pastes during the storage time. The results of variance analysis of the data related to pH changes showed a significant effect of the treatments in different concentrations (*p* < .05) and a significant effect of the storage time on the pH level (*p* < .05). The analysis of the data showed that with the increase in the amount of bacteria in the formulation, the pH decreased, and this decrease was less in the combined treatments with 0.3% propionate than in the single treatments. Over time, the pH has decreased. In fact, the increase in the amount of bacteria and the production of lactic acid during the storage period has caused a significant decrease in the pH measured in the pastes. As the obtained results show, with the increase in the amount of bacteria added to the paste, the pH of the paste decreased compared to the control sample, which is due to the production of lactic acid by these bacteria, which is in agreement with the results obtained by other researchers (Sadeghi et al., 2008). On the other hand, the pH of combined treatments with calcium propionate or calcium propionate alone was higher than the treatments containing lactic acid bacteria alone and was close to the control sample, which according to the results of Bolourian et al. (2010) is probably due to the effect of these two substances together act as a buffer system and prevent a significant change in pH, which was consistent with the results of Abu‐Ghoush et al. (2008). Many of the beneficial properties attributed to sourdough are determined by the acidifying activity of lactic acid bacteria. Acid production depends on factors such as fermentation temperature, time, and paste performance. In general, higher temperature, higher water content of sourdough, and use of whole flour increases the production of acids in sourdough (Sadeghi et al., 2008).

The results of analysis of variance of the data showed the significant effect of the treatments in different concentrations and the significant effect of the storage time on the minimum amount of crude protein of the pastes (*p* < .05). Comparing the average results of measuring the minimum crude protein of yufka paste based on Duncan's test (Table 4) showed that the minimum crude protein decreased over time in different treatments and the highest amount was related to the 1st and 5th days. Among the treatments, the highest amount of protein was related to the control sample and the sample containing only chemical preservative. The importance of leavening bacteria is that they can consume the carbohydrates in flour and also the fermented protein product for their metabolism and in this way produce lactic acid and acetic acid which are effective for forming and preparing paste and baking. The decrease in the amount of protein observed with the increase in the amount of added bacteria and also the increase in time, according to the results of Bolourian et al. (2010), is probably due to the presence of proteolytic enzymes in the sourdough system and the breakdown of different proteins in the paste, turning them into smaller substances such as free amino acids.

**TABLE 4:** Comparison of the average results of pH and protein in different treatments of yufka paste during storage time.

Treatment	Characteristics	Day 1	Day 5	Day 10	Day 15	Day 20
1	pH	5.46 ± 0.67^Aa^	4.98 ± 0.79^Aa^	5.28 ± 0.29^Aa^	4.51 ± 1.35^Aa^	3.72 ± 0.13^Aa^
	Protein	8.42 ± 0.47^Ab^	8.81 ± 0.07^Ab^	7.75 ± 0.20^Bb^	7.27 ± 0.13^Bb^	6.82 ± 0.30^Bb^
2	pH	5.19 ± 1.17^Ac^	3.99 ± 0.12^Ac^	4.04 ± 0.87^Ac^	4.15 ± 0.80^Ac^	3.52 ± 0.07^Ac^
	Protein	8.12 ± 0.61^Ac^	8.18 ± 0.37^Ac^	7.28 ± 0.43^Bc^	7.14 ± 0.29^Bc^	7.01 ± 0.19^Bc^
3	pH	4.81 ± 0.39^Ad^	4.66 ± 0.14^Ad^	4.61 ± 0.13^Ad^	4.57 ± 0.16^Ad^	4.41 ± 0.25^Ad^
	Protein	9.11 ± 0.48^Ab^	8.24 ± 0.69^Ab^	7.56 ± 0.09^Bb^	7.70 ± 1.28^Bb^	7.76 ± 0.19^Bb^
4	pH	5.17 ± 0.23^Abc^	5.06 ± 0.15^Abc^	4.33 ± 0.08^Abc^	3.66 ± 0.18^Abc^	3.51 ± 0.48^Abc^
	Protein	9.24 ± 0.64^Aab^	9.05 ± 0.31^Aab^	8.11 ± 0.14^Bab^	6.92 ± 0.14^Bab^	6.79 ± 0.15^Bab^
5	pH	5.44 ± 0.29^Abc^	5.21 ± 0.17^Abc^	4.94 ± 0.05^Abc^	4.18 ± 0.36^Abc^	4.01 ± 0.31^Abc^
	Protein	7.94 ± 0.41^Ac^	7.77 ± 0.09^Ac^	7.46 ± 0.44^Bc^	6.99 ± 0.55^Bc^	6.68 ± 0.46^Bc^
6	pH	5.32 ± 0.08^Abc^	5.18 ± 0.48^Abc^	4.44 ± 0.53^Abc^	4.38 ± 0.23^Abc^	4.29 ± 0.22^Abc^
	Protein	8.98 ± 0.48^Aab^	9.01 ± 0.54^Aab^	8.84 ± 0.40^Bab^	8.29 ± 0.74^Bab^	7.31 ± 0.14^Bab^
7	pH	5.89 ± 0.77^Aab^	5.72 ± 0.27^Aab^	5.67 ± 0.09^Aab^	4.79 ± 0.13^Aab^	4.73 ± 0.10^Aab^
	Protein	8.97 ± 1.16^Aab^	8.88 ± 0.23^Aab^	8.68 ± 1.08^Bab^	8.46 ± 0.53^Bab^	8.23 ± 0.20^Bab^
Control	pH	6.07 ± 0.04^Abc^	5.96 ± 0.18^Abc^	5.83 ± 0.08^Abc^	4.77 ± 0.14^Abc^	4.69 ± 0.09^Abc^
	Protein	10.18 ± 0.59^Aa^	9.78 ± 0.25^Aa^	8.89 ± 0.72^Ba^	8.83 ± 0.13^Ba^	8.86 ± 0.39^Ba^

*Note*: Similar lowercase letters depending on the treatment (row) and uppercase letters depending on the time (column) indicate no significant difference at the 5% probability level.

### 3.3 The survival of *Lactobacillus plantarum* and molds

Table 5 shows the comparison of the average results of *L. plantarum* bacteria viability and mold count in different treatments of yufka pastes during the storage time. The results of the analysis of variance of the data related to the survival rate of *L. plantarum* showed that the effect of different treatments on survival was statistically significant (*p* < .05), the storage time also showed a significant effect on the survival of this bacterium (*p* < .05). Also, the comparison of average data using Duncan's method showed the highest survival rate in treatments containing 10^3^ cfu/g of *L. plantarum* bacteria and treatments containing 10^3^ cfu/g *L. plantarum* bacteria combined with 0.3% calcium propionate and the lowest rate related to higher concentration of bacteria. In the examination of the time, the lowest and highest survival rates were related to the first day and the 20th day, respectively. In general, with increasing time, the survival rate of *L. plantarum* bacteria increased in different treatments of yufka paste.

**TABLE 5:** Comparison of the average results of *Lactobacillus plantarum* bacteria viability and mold count in different treatments of yufka pastes during storage time.

Treatment	Characteristics	Day 1	Day 5	Day 10	Day 15	Day 20
1	Bacterial survival rate	4.10 ± 0.14^Bab^	4.74 ± 0.03^Bab^	4.87 ± 0.42^Bab^	5.49 ± 0.12^Bab^	6.05 ± 0.55^Aab^
	Mold count	1.61 ± 0.94^Acd^	1.33 ± 0.47^Acd^	1.00 ± 0.82^Acd^	0.47 ± 0.33^Acd^	0.47 ± 0.23^Acd^
2	Bacterial survival rate	4.70 ± 0.00^Bab^	4.82 ± 0.09^Bab^	4.90 ± 0.04^Bab^	4.76 ± 0.05^Bab^	6.06 ± 0.59^Aab^
	Mold count	0.47 ± 0.38^Aef^	ND^Aef^	1.33 ± 0.47^Aef^	0.37 ± 0.25^Aef^	ND^Aef^
3	Bacterial survival rate	5.25 ± 0.44^Bab^	5.10 ± 0.14^Bab^	4.93 ± 0.48^Aab^	5.43 ± 0.31^Bab^	5.29 ± 0.54^Aab^
	Mold count	1.39 ± 0.47^Af^	1.27 ± 0.47^Af^	0.97 ± 0.44^Af^	0.67 ± 0.47^Af^	0.59 ± 0.47^Af^
4	Bacterial survival rate	4.77 ± 0.33^Bb^	4.91 ± 0.08^Bb^	5.77 ± 0.33^Bb^	5.95 ± 0.10^Bb^	6.17 ± 0.47^Ab^
	Mold count	0.47 ± 0.36^Adef^	0.47 ± 0.33^Adef^	0.47 ± 0.33^Adef^	0.47 ± 0.32^Adef^	1.25 ± 0.13^Adef^
5	Bacterial survival rate	4.80 ± 0.03^Bab^	5.08 ± 0.29^Bab^	4.94 ± 0.05^Bab^	5.17 ± 0.11^Bab^	5.14 ± 0.048^Aab^
	Mold count	1.00 ± 0.00^Ac^	0.47 ± 0.35^Ac^	0.47 ± 0.33^Ac^	0.49 ± 0.27^Ac^	0.94 ± 0.23^Ac^
6	Bacterial survival rate	4.03 ± 0.41^Ba^	4.84 ± 0.72^Ba^	5.79 ± 0.77^Ba^	5.82 ± 0.09^Ba^	5.03 ± 0.46^Aa^
	Mold count	1.00 ± 0.00^Acde^	1.07 ± 0.47^Acde^	1.13 ± 0.82^Acde^	0.94 ± 0.93^Acde^	0.82 ± 0.61^Acde^
7	Bacterial survival rate	–	–	–	–	–
	Mold count	2.33 ± 1.70^Ab^	2.67 ± 0.47^Ab^	1.33 ± 0.047^Ab^	1.22 ± 0.94^Ab^	1.17 ± 0.47^Ab^
Control	Bacterial survival rate	–	–	–	–	–
	Mold count	3.87 ± 0.82^Aa^	3.97 ± 0.47^Aa^	4.93 ± 1.25^Aa^	5.00 ± 0.82^Aa^	4.97 ± 0.47^Aa^

*Note*: Similar lowercase letters depending on the treatment (row) and uppercase letters depending on the time (column) indicate no significant difference at the 5% probability level.

Abbreviation: ND, not detected.

The results of analysis of variance of the mold count data showed a significant effect of the treatments in different concentrations on the count of molds (*p* < .05), but the storage time did not have a significant effect on the amount of mold grown on the pastes (*p* > 0.05). The comparison of the average data related to the amount of mold growth in 0.01 dilution based on Duncan's test is shown in Table 5. As can be seen, among the different treatments, the lowest amount of mold was related to samples containing *L. plantarum* bacteria alone, especially in concentrations of 10^3^ and 10^5^ cfu/g, and the highest amount was related to the control sample. Over time, the amount of mold in the samples containing the preservative decreased, while in the control sample, the amount of mold also increased with the increase of time. These results were consistent with the results obtained in the research of Sadeghi et al. (2008), Tarar et al. (2010), and Akbari et al. (2013). The results showed inhibition of growth and prevention of mold growth during storage, especially when *L. plantarum* was used alone and in combination with calcium propionate, which is consistent with the results obtained by Dal Bello et al. (2007). The mold count in the combined use of *L. plantarum* bacteria and 0.3% calcium propionate was lower than the single use of the chemical preservative. Anyway, this result shows the synergistic effect of green preservative and chemical preservative in controlling moldy spoilage. This finding was in line with the research of Tarar et al. (2010) as they showed that the combined use of preservatives (calcium propionate along with acetic acid or lactic acid) significantly reduces the growth of mold over time and increases the shelf life of the product due to the synergistic effect.

### 3.4 Sensory characteristics

**FIGURE 2:**
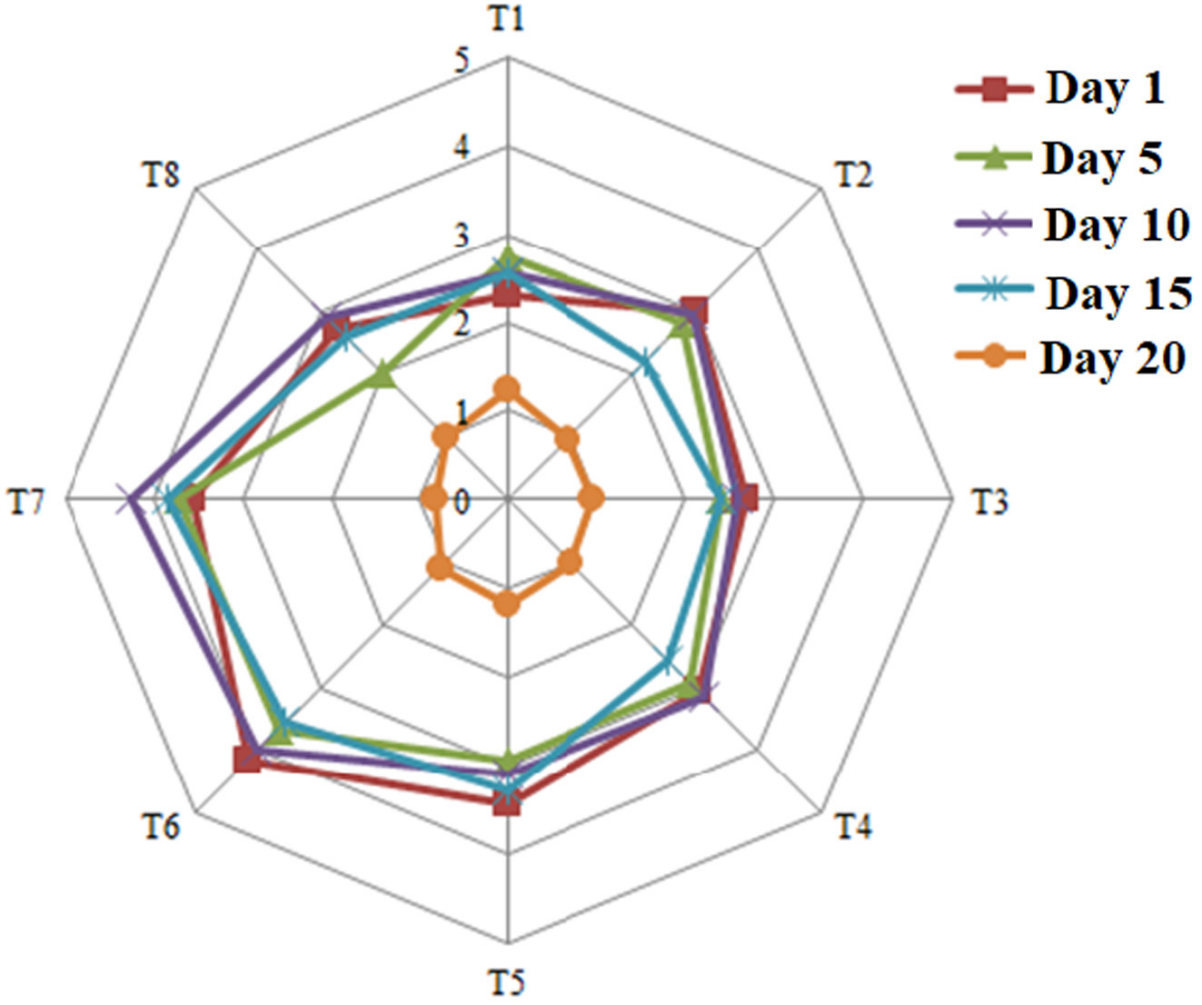
Spider diagram of sensory evaluation (overall acceptance) of yufka paste samples during storage time.

The samples of yufka paste prepared by 20 evaluators were examined in terms of color, appearance, aroma, and flexibility and were scored as acceptability and general acceptability from 1 for the weakest sample to 5 for the best sample. The results (general acceptability) obtained on different days of storage for the eight treatments prepared are shown in Figure 2. As can be seen, the highest scores until day 15 were related to samples containing 10^7^ cfu/g of bacteria combined with 0.3% propionate (treatment 6). On the 20th day, the retention of points decreased drastically. On the 20th day, the highest score was related to the sample containing 103 cfu/g of bacteria (treatment 1). Therefore, it can be said that with the use of green preservative, in addition to increasing the shelf life of the product and reducing the amount of mold, in terms of sensory and general acceptability, the product has obtained an acceptable score, which, in addition to increasing the shelf life of the product, can prevent the loss of the product, improve its sales, and increase the economic efficiency of the product.

### 3.5 Modeling the antimold effect of *Lactobacillus plantarum*

**FIGURE 3:**
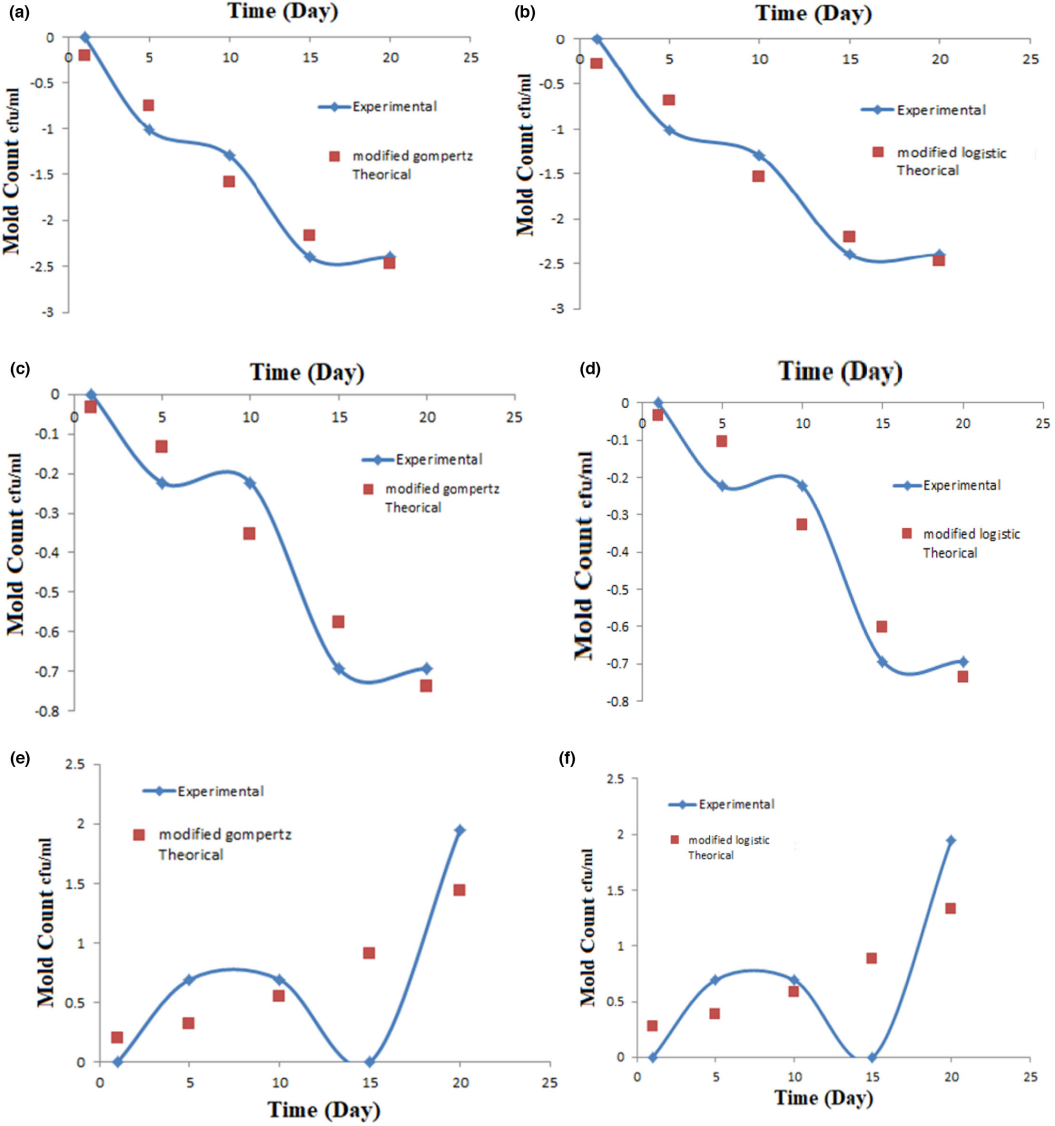
Diagrams of modified Gompertz model (a) and modified logistic model (b) in treatments containing 10^3^ cfu/mL *Lactobacillus plantarum* bacteria, diagrams of modified Gompertz model (c) and modified logistic model (d) in treatments containing 0.3% of calcium propionate, and the graphs of modified Gompertz model (e) and modified logistic model (f) in treatments containing 10^3^ cfu/mL *L. plantarum* bacteria and 0.3% of calcium propionate compared to mold growth.

Figure 3 shows graphs of modified Gompertz model and modified logistic model in treatments containing 10^3^ cfu/mL *L. plantarum* bacteria, graphs of modified Gompertz model and modified logistic model in treatments containing 0.3% calcium propionate, and graphs of modified Gompertz model and modified logistics model in treatments containing 10^3^ cfu/mL of *L. plantarum* bacteria and 0.3% of calcium propionate with respect to growth of mold. Also, Table 6 shows the data obtained during the relevant experiments in MATLAB software at different concentrations and times using the mentioned models.

**TABLE 6:** Relevant experiments in MATLAB software at different concentrations and times using four different models.

Treatment type	Model type	Correlation coefficient (*R* ^2^)	Root mean square error	Chi‐square
10^3^ cfu/mL *Lactobacillus plantarum*	Modified Gompertz	.969	0.223	0.062
	Modified logistic	.966	0.235	0.029
	Gompertz theory	−.949	1.229	1.888
	Logistic theory	−.949	1.229	1.888
3% Calcium propionate	Modified Gompertz theory	.945	0.091	0.010
	Modified Logistic theory	.953	0.084	0.009
	Gompertz theory	−.722	0.646	0.522
	Logistic theory	−.722	0.646	0.522
10^3^ cfu/mL *L. plantarum +* 3% Calcium propionate	Modified Gompertz theory	.707	0.506	0.320
	Modified logistic theory	.707	0.517	0.334
	Gompertz theory	.820	0.428	0.229
	Logistic theory	.843	0.404	0.204

Modeling the number of molds over time with the presence of *L. plantarum* in yufka paste was determined and the used models (Gompertz and Logistic and modified Gompertz and modified Logistic) were able to predict the antimold effects of *L. plantarum*. According to the results, the growth of mold in the treatments containing bacteria at a lower concentration is better than the treatments containing bacteria and propionate or only propionate or the control treatment. According to the analysis of the modeling results, with the passage of time in the presence of *L. plantarum*, the speed of mold growth decreases with the increase in the concentration of bacteria until the environment becomes acidic, but as the environment becomes more acidic, the number of *L. plantarum* decreases and its effectiveness also decreases. Therefore, in lower concentrations, due to less acid production, we saw more effect.

As can be seen in Table 6, four models were used for prediction in all treatments. The results show that in yufka pastes prepared with a lower concentration, i.e., 10^3^ cfu/mL of *L. plantarum* bacteria, 10^3^ cfu/mL of *L. plantarum* bacteria combined with 0.3 propionate and 0.3 propionate, two modified Gompertz and modified logistic models due to having a higher correlation coefficient and less root mean square error than other models is able to express the kinetic form of total mold count. As can be seen in the graphs, in the shown concentrations and treatments, two modified Gompertz and modified logistic models have made appropriate predictions. Therefore, by modeling the laboratory data and examining the graphs obtained in the investigation of the antimold effects of *L. plantarum* on the total count of mold and yeast, it was concluded that the effect of time, concentration, and type of treatment on the count of mold are significant, and modified Gompertz and modified logistic models are the best model for predicting the total count in the product at any time.

## 4 CONCLUSION

The effect of single and combined use of chemical (calcium propionate) and biological (*L. plantarum*) preservatives on increasing the shelf life of yufka by taking into account its physicochemical and microbial characteristics was investigated and according to the results obtained, by increasing the concentration of bacteria and using it together with propionate, the amount of bacteria was increased in the product. Increasing the storage time increased the viability of bacteria and increased the number of counts. With the increase in the amount of bacteria in the formulation, the pH decreased. Over time, the pH of samples decreased. The increase in the amount of bacteria and lactic acid production during the storage period caused a significant decrease in the pH measured in the paste. The count of mold and yeast in the combined use of *L. plantarum* bacteria and 0.3% propionate was lower than the single use of propionate chemical preservative. On the 20th day of storage, sensory scores decreased sharply, and the highest score was related to the sample containing 10^3^ cfu/mL bacteria. According to the modeling results, the lower the concentration of *L. plantarum* bacteria, the lesser the molds in the sample grew. According to the results, the growth of mold in the treatments containing bacteria at a lower concentration is better than the treatments containing bacteria and propionate or only propionate or the control treatment. In yufka pastes prepared with a lower concentration, that is, 10^3^ cfu/mL of *L. plantarum* bacteria, 10^3^ cfu/mL of *L. plantarum* bacteria combined with 0.3 propionate and 0.3 propionate, two modified Gompertz and modified logistic models due to having a higher correlation coefficient and the less root mean square error compared to other models are able to express the kinetic form of total mold count. In general, it can be said that the use of green preservatives can reduce the amount of chemical preservatives used to increase the shelf life of bread products, and it can also improve the sensory properties and overall acceptance of the product while maintaining the physicochemical characteristics over time.

## AUTHOR CONTRIBUTIONS

**Hannaneh Bahmanpour:** Conceptualization (equal); data curation (equal); investigation (equal); methodology (equal); software (equal). **Mahmood Sowti Khiabani:** Conceptualization (equal); formal analysis (equal); validation (equal). **Sajad Pirsa:** Confirming article data and writing and editing the article.

## FUNDING INFORMATION

The author(s) received no financial support for the research, authorship, and/or publication of this article.

## CONFLICT OF INTEREST STATEMENT

There is no conflict of interest between authors.

## Data Availability

Research data are not shared.
